# ‘Earlier than Early’ Detection of Breast Cancer in Israeli *BRCA* Mutation Carriers Applying AI-Based Analysis to Consecutive MRI Scans

**DOI:** 10.3390/cancers15123120

**Published:** 2023-06-08

**Authors:** Debbie Anaby, David Shavin, Gali Zimmerman-Moreno, Noam Nissan, Eitan Friedman, Miri Sklair-Levy

**Affiliations:** 1 Department of Diagnostic Imaging, Sheba Medical Center, Ramat Gan 52621, Israel; 2 Sackler Faculty of Medicine, Tel-Aviv University, Tel-Aviv 6910201, Israel; 3 Meirav High Risk Center, Sheba Medical Center, Ramat Gan 52621, Israel

**Keywords:** BRCA, early diagnosis, artificial intelligence, breast cancer, MRI

## Abstract

**Simple Summary:**

Women who have a genetic mutation of BRCA1 or BRCA2 are at a significantly higher risk for developing breast cancer. Early detection is crucial for an improved prognosis, therefore they are offered an intensive follow-up program, including a yearly MRI scan. Although MRI is the most sensitive imaging modality for breast cancer detection, it was found that a significant number of tumors are overlooked or misinterpreted, leading to a delayed diagnosis. Aiming to improve breast cancer diagnosis at early stages, we developed an artificial-intelligence based tool that is shown to classify correctly ~65% of the tumors at an early time point. These tumors were not suspected/diagnosed by the radiologists at that time point, but only at the next MRI scan. We believe that such an AI-system could serve as an aid to radiologists, improve their decision-making and achieve an ‘earlier than early’ diagnosis of breast cancer in BRCA carriers.

**Abstract:**

Female *BRCA1/BRCA2* (=*BRCA*) pathogenic variants (PVs) carriers are at a substantially higher risk for developing breast cancer (BC) compared with the average risk population. Detection of BC at an early stage significantly improves prognosis. To facilitate early BC detection, a surveillance scheme is offered to *BRCA* PV carriers from age 25–30 years that includes annual MRI based breast imaging. Indeed, adherence to the recommended scheme has been shown to be associated with earlier disease stages at BC diagnosis, more in-situ pathology, smaller tumors, and less axillary involvement. While MRI is the most sensitive modality for BC detection in *BRCA* PV carriers, there are a significant number of overlooked or misinterpreted radiological lesions (mostly enhancing foci), leading to a delayed BC diagnosis at a more advanced stage. In this study we developed an artificial intelligence (AI)-network, aimed at a more accurate classification of enhancing foci, in MRIs of *BRCA* PV carriers, thus reducing false-negative interpretations. Retrospectively identified foci in prior MRIs that were either diagnosed as BC or benign/normal in a subsequent MRI were manually segmented and served as input for a convolutional network architecture. The model was successful in classification of 65% of the cancerous foci, most of them triple-negative BC. If validated, applying this scheme routinely may facilitate ‘earlier than early’ BC diagnosis in *BRCA* PV carriers.

## 1. Introduction

*BRCA1* and *BRCA2* (heretofore *BRCA*) germline pathogenic variant (PVs) carriers are at a significantly higher risk for breast cancer (BC), with estimated cumulative lifetime risk of 55% to 65% and 45% to 47%, respectively [1,2]. *BRCA1* carriers commonly develop aggressive cancers (triple-negative with high nuclear grade), with peak incidence in the 41–50 age-group, while *BRCA2* carriers commonly develop cancers that are less aggressive (estrogen receptor (ER)-positive) with peak incidence in the 51–60 age-group [3,4]. Tumor volume doubling time in invasive BCs is reportedly twice as high in *BRCA* carriers (46 and 52 days in *BRCA1* and *BRCA2*, respectively) compared with age adjusted *BRCA* wild type high-risk women [5]. Thus, within 1 year, a tumor may double its volume by 4–5 times, implying a doubling of tumor diameter, a fact that may adversely affect tumor stage at diagnosis and consequent prognosis. Therefore, early detection of BC is crucial for diagnosis of curable non-metastatic disease, at a stage in which the cancer cells have not yet gained the ability to metastasize and become resistant to targeted therapies [6]. 

The current screening guidelines for female *BRCA* PVs carriers, including bi-annual breast imaging [7], have been shown to increase the rate of early diagnosis with more in-situ pathology, smaller tumors, and less axillary involvement compared with carriers who do not adhere to the suggested surveillance scheme [7,8,9,10]. MRI is considered the most sensitive modality for BC detection in *BRCA* PV carriers, with sensitivity rates of up to 96% [10,11,12,13]. Yet several studies have shown that, in retrospective analyses, in a significant number of MRI detected BCs (50–74%), localized radiological abnormalities were present in the prior MRI, which was performed approximately one year before actual BC diagnosis [12,14,15,16,17]. Pages et al. [18] and Seo et al. [19] reported that in ~50% of the reviewed cases (from a total of 56 and 72 consecutive pairs of MR imaging studies, respectively) a suspicious radiological finding of ~1 cm in size could be retrospectively visualized in a previously performed MRI. Notably, Gubern-Merida et al. [20] reported rates of 60% (24/40 pairs of MRIs) displaying misclassified pre BC diagnosis MRI abnormal findings, and Bilocq-Lacoste et al. [17] reported even higher rates of 74% (57/77 pairs of MRIs). The false negative pre-BC diagnosed MRIs in these studies were attributed to several factors: resemblance of the cancerous lesion to physiologic enhancement, small lesion size, stability in size and location of a non-mass in a postsurgical area [18,19]. Even when small lesions (usually 5 mm in size) are detected by MRI, their classification may be challenging because their morphologic and kinetic characteristics are commonly benign [21]. 

Recently, artificial intelligence (AI) algorithms are being assessed as additional tools for BC screening and diagnosis, mainly based on mammography captured data analyses [22,23,24,25,26,27,28,29]. MRI-based AI algorithms are also being evaluated for BC diagnosis, improved specificity, treatment response assessment, and anatomic segmentations of fibro-glandular tissue (FGT), allowing the quantification of background parenchymal enhancement (BPE) [30,31,32,33,34]. One of the major challenges of MRI breast imaging analyses is that of characterizing sub-centimeter lesions, which often display benign appearing features, and differentiating them from abundant types of enhancing foci, for either benign etiologies or focal background parenchymal enhancement (BPE). With that in mind, we developed and clinically-tested an AI network, trained to correctly classify enhancing foci detected on consecutive breast MRI studies of *BRCA* PV carriers, in order to facilitate ‘earlier than early’ BC diagnosis. 

## 2. Materials and Methods

### 2.1. Study Population

This study was approved by the institutional review board and the need to obtain a written informed consent was waived, given the study outline and retrospective nature. 

A retrospective search was performed in the dataset of the Meirav high-risk Clinic repository at Sheba Medical Center for all *BRCA* PV carriers with MRI detected BC and a prior MRI examination in the preceding 18 months, between 2012–2021. Diagnosis was based on biopsy obtained pathological report and the location of the tumor was determined by MRI. Cancer-free *BRCA* PV carriers seen at the same clinic during the study period with consecutive MRIs and with at least one year clinical and radiological follow-up were retrieved and served as controls. 

Breast MRI was performed at 1.5 Tesla (Signa Excite HDX, GE Healthcare, Chicago, IL, USA) using a dedicated double breast coil equipped with eight channels. Dynamic contrast enhanced (DCE)-MRI protocol was obtained via axial vibrant multiphase 3D DCE T1-weighted sequence, prior and four times after an automated injection of contrast agent bolus (0.1 mL/kg at 2 mL/s Dotarem, (gadoterate meglumine, Guebet)), followed by a 20 mL saline flush. The first post-contrast images were centered at 1:25 min after injection and the rest were centered every 2 min following the first images, such that the delayed images were centered at 7:35 min after injection. The parameters used for the DCE-MRI were as follows: echo time (TE) = 2.6 ms, repetition time (TR) = 5.4 ms, flip angle = 15°, bandwidth = 83.3 kHz, matrix = 512 × 364, field of view (FOV) = 340 mm and slice thickness = 2 mm. 

The pre-contrast images were subtracted from the 2nd time-point post-contrast images to create sub-DCE images. These were collected from the prior MRIs of all patients.

### 2.2. MR Images, Clinical, Radiological and Pathological Data Analysis

Patients’ consecutive MRIs; ‘prior’ (~1 year before diagnosis) and ‘diagnosis’ were collected along with their clinical data, pathology of the diagnosed tumors and image radiology features, including age, time between consecutive scans, exact *BRCA* PV, breast imaging reporting and data system (BI-RADS) score, and BPE grade in prior scans, tumor size at diagnosis, tumor histopathological type, histological grade and immunohistochemistry results for hormonal receptor expression. Lesions at diagnosis and abnormalities in the prior scans (if existed) were retrospectively morphologically characterized (focus, mass, non-mass) according to the BI-RADS lexicon (acr.org/birads) by an experienced breast radiologist (MSL).

### 2.3. Lesion Segmentation and Morphological/Kinetic Assessment

The prior MRIs were used for analysis and the diagnosis MRIs and biopsies served as ground-truth for cancer/cancer-free labeling and for identification of the tumor area and its delineation.

Manual segmentation was performed on picture archiving and communication system (PACS). In the cancer patients, the central slice of the tumor was manually marked on the sub-DCE image of the diagnosis MRI by DA in agreement with radiologist MSL. Manual co-registration between the two consecutive scans in each patient was performed in PACS and then the corresponding region of the known cancerous tumor was identified in the prior scan based on anatomical landmarks. In cases in which the region comprised of an enhancing abnormality, its central slice was manually marked. In the cancer-free women, if the prior MRIs had a reported lesion/abnormality, it was marked for control. Otherwise, a prominent enhancing focus (regarded as BPE) was chosen and marked. The corresponding regions in the following MRI scans were also carefully identified and marked, if existed. In all cases, the segmentation was copied from the subtraction image to the raw data including the pre-contrast, the first and the fourth post-contrast time point images. Then, kinetic features were calculated according to:

(1) $$Initial\;enhancement=\frac{\left(SE\;in\;1st\;time\;point\right)-(SE\;in\;pre\;contrast)}{SE\;in\;pre\;contrast}*100$$

(2) $$Delayed\;phase=\frac{\left(SE\;in\;4th\;time\;point\right)-(SE\;in\;1st\;time\;point)}{\;SE\;in\;1st\;time\;point}*100$$

where SE stands for signal enhancement. Initial enhancement was categorized as ‘slow’ when smaller than 50%, ‘medium’ when between 50–100% and ‘fast’ when larger than 100%. Delayed phase was defined as ‘persistent’ for a larger increase than 10%, ‘plateau’ when between a decrease of 10% and an increase of 10%, and ‘washout’ for a decrease of more than 10% [35]. 

Segmented lesions and abnormalities in both prior and diagnosis MRIs were morphologically characterized into 3 categories: focus, mass and non-mass, according to BI-RADS lexicon (acr.org/birads). Lesion size was manually measured in PACS based on the largest diameter. 

### 2.4. Convolutional Neural Network (CNN) Architecture 

**Figure 1:**
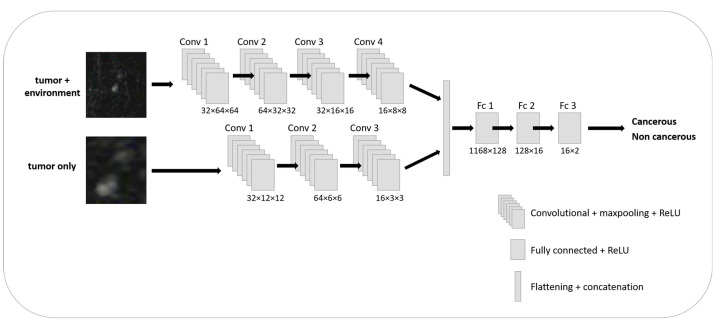
The proposed network architecture with input of 2 ROIs per lesion from a sub-DCE 2D image.

The model was categorized between tumor and non-tumor ROIs. Two patches per abnormality or BPE regions served as input: (1) the regions immediately around the abnormality or BPE (resized to 24 × 24) and (2) a larger field of view (FOV) region including the abnormality’s or BPE’s (128 × 128) surroundings. This allowed capture of both global spatial information and smaller local details [36]. The proposed network architecture, illustrated in Figure 1, included a repeated application of a 3 × 3 convolution, followed by a rectified linear activation function (ReLU) and a 2 × 2 max pooling operation with stride 2 for down-sampling for feature extraction [37]. This process was repeated separately for both inputs described above. Then, the feature vectors were flattened and concatenated into a single feature vector. This then served as input for a multi-layer-perceptron classifier and finally pushed the output through a soft-max activation to provide a valid distribution. The training was performed using an Adam optimizer with a learning rate of 1 × 10^−4^ that minimizes a binary cross entropy loss function. The feature extractors were pre-trained on a Cifar-19 dataset for an improved initialization of the model’s weights. Cross-validation was performed with a leave-one-out procedure.

### 2.5. Statistics

The data were statistically analyzed using SPSS 29.0 (Chicago, IL, USA). *T*-tests or Chi-square tests were used to assess differences between independent groups. Repeated measures ANOVA with a Greenhouse-Geisser correction was used to test the change in lesion characteristics between prior and diagnosis scans in the cancerous and non-cancerous groups. Time of scan was used as the within-subject factor and group as the between-subjects factor. Paired t-tests were applied as post-hoc analysis in the cases where a significant interaction between time and group was found. Significance was set at *p* < 0.05.

## 3. Results

Overall, the study group encompassed 53 biopsy-proven BCs in *BRCA* PV carriers with a pre diagnosis MRI available for analysis (367.6 days between scans on average). As controls, 53 cancer-free *BRCA* PV carriers with two consecutive MRIs performed during the study period were analyzed. 

**Figure 2:**
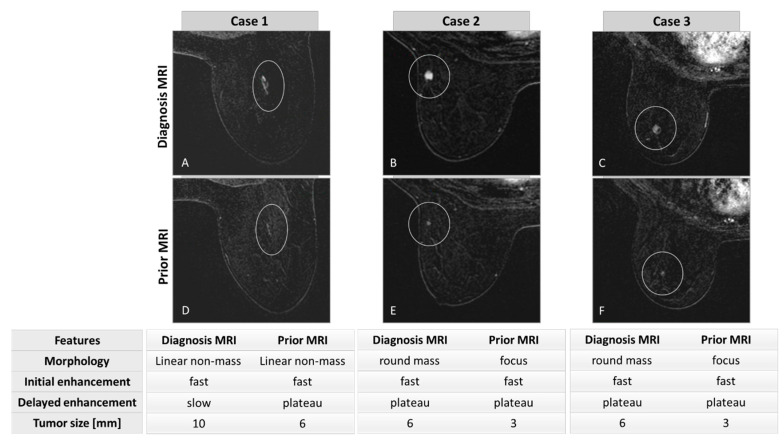
Diagnosed BCs in three patients (top row) and their corresponding radiological abnormalities as appearing in the previous MRIs (bottom row). Tumors and abnormalities are marked by a white circle. The morphological and kinetic features are shown below the images. Case 1–74 years old *BRCA1* PV carrier, diagnosed with grade 3 triple negative ductal carcinoma in situ (DCIS) (**A**). Linear non mass enhancement at the prior MRI (**D**) was unchanged throughout several previous MRIs (not presented), and therefore reported as BI-RADS 2. Case 2–64 years old *BRCA1* PV carrier, diagnosed with grade 3 triple negative invasive ductal carcinoma (IDC) (**B**). Enhancing focus at the prior MRI was misinterpreted as intramammary lymph node and reported as BI-RADS 2 (**E**). Case 3–67 years old *BRCA1* PV carrier, diagnosed with grade 3 triple negative IDC (**C**). Enhancing focus at the prior MRI was not detected by the radiologist and reported as BI-RADS 2 (**F**).

In retrospective visualization, a radiological abnormality could be detected in previous MRIs of 32/53 BC patients (60.4%) in the same anatomical region where a tumor was subsequently located. Figure 2 presents consecutive MRIs of three representative BC cases (Figure 2A–C) in whom an abnormality could be visualized in their prior MRI (Figure 2D–F). The time difference between the two scans was 357, 418, and 387 days, respectively.

Radiological features and tumor pathological characteristics of all 53 BC cases are shown in Table 1. Of 32 patients in whom a radiological abnormality was present in previous MRI imaging, five (15.6%) cases were reported as BI-RADS 0 due to that abnormality or due to dense breasts, indicating that more imaging information is required (mammography and ultrasound) in order to make an informed medical decision. Of the five patients, three followed the recommendations, and the findings were either determined as post-surgical changes or were not detected, thus were not diagnosed at that time point. 8/32 (25%) patients were reported as BI-RADS 3 due to unchanged findings, post-surgical changes, dense breasts or benign-looking lesions. The rest (59.4%) were reported as radiologically negative (BI-RADS 1 or 2 on prior scan). Age at diagnosis, distribution of *BRCA* PV, days between the two scans, BI-RADS and BPE in the prior scan were similar between the case group (those with BC) and the cancer-free group.

Invasive ductal carcinoma (IDC) tumors were more common in both groups compared with ductal carcinoma in-situ (DCIS), while a large number were high grade triple negative (44% and 60% of patients with abnormality and without abnormality in the prior MRI, respectively). Tumor size at diagnosis was significantly different between the groups (student’s *t*-test, *p* = 0.01), while patients who had an abnormality in the early scan presented with larger tumor sizes than those who did not at the diagnosis MRI (12.8 mm vs. 7.8 mm on average, respectively). The distribution of BPE in the prior scans was different between the groups: in patients who had an abnormality on the prior scan, BPE was distributed evenly between minimal-mild and moderate-marked whereas, in those who did not show an abnormality more MRIs showed a minimal-mild BPE score than moderate-marked (71.4% vs. 28.6%, respectively). Yet this difference was not statistically significant (*p* = 0.08). 

Age of the individuals diagnosed with BC and age at analysis for the cancer-free group, days between the consecutive scans, BIRADS and BPE distributions were similar between these two groups. Mutated *BRCA* gene distribution was statistically significantly different (*p* = 0.015): most cancer patients were *BRCA1* PV carriers (73.6%) whereas in the cancer-free cohort an almost even representation was seen for both genes. 

Lesion characteristics were compared between the two consecutive scans in the cancer and cancer-free patients (Table 2). The overall mean difference of the lesion size over time was statistically significant (F(1,59) = 23.742, *p* < 0.001), as was the interaction time*group (F(1,59) = 25.990, *p* < 0.001). Additionally, the overall mean difference of the morphology characteristics over time (focus, mass, non-mass) was statistically significant (F(1,63) = 5.968, *p* = 0.017) as was the interaction (F(1,63) = 13.871, *p* < 0.001). A paired *t*-test revealed significance between these features in the cancer group, only showing smaller lesion size in the prior scans compared with the diagnosis scans (6.1 mm vs. 10.8 mm on average, *p* < 0.0001), most of which were determined to be ‘focus’ (59.3%). The morphology type distribution was significantly different between the prior and diagnosis scans in the cancer group (*p* = 0.0001), showing mostly ‘mass’ lesions at diagnosis (56.3%). 

In the cancer-free cohort, almost 40% of the enhancements that were detected in the prior scans did not appear in the follow-up MRI. In cases where lesions were visualized in both consecutive MRIs, lesion size and morphology were similar. 

Kinetic characteristics were similar between the consecutive scans in both the cancer and cancer-free groups. 

**Figure 3:**
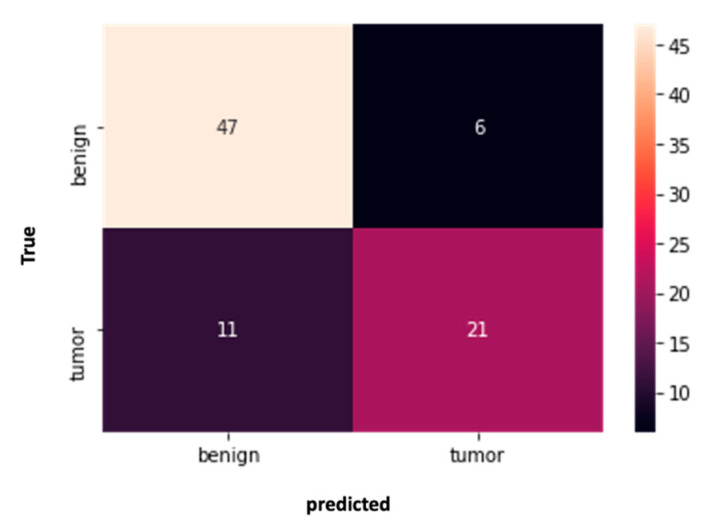
Confusion matrix of the performance of the network for classification of very early lesions/abnormalities in prior MRI scans to diagnosis scans of breast cancer.

Analysis based on an AI network architecture was used to achieve early detection of the abnormalities on prior MRIs. The network was based on a total of 85 MRI scans: prior MRIs of the 32 cancer patients who had an abnormality and prior scans of the 53 cancer-free patients. The network successfully classified 21/32 cancer cases (65.6%) as cancerous and 47/53 cancer-free cases (88.7%) as non-cancerous, as shown in Figure 3. 

MR scan characteristics and tumor pathology and morphology were observed for the 21 successfully classified cancer cases, compared with the 11 unsuccessful classified cases, by the network (Table 3 and Table 4).

Interestingly, the molecular subtype distribution was significantly different between the groups (*p* = 0.016). In the successfully classified group of patients, most tumors were triple negative (61.9%), while in the unsuccessfully classified patients, most tumors were HR+/HER2− (50%). The tumor size showed a trend *p*-value of 0.05, while the correctly classified abnormalities in the prior scans had a smaller diameter compared with those which were not classified correctly (10.6 mm vs. 16.7 mm, respectively).

Morphology and kinetic characteristics were similar in both the successfully and unsuccessfully classified groups of patients. In both groups, most abnormalities were ‘focus’, with a medium/fast initial enhancement and persistent delayed phase. Computer aided detection (CAD) was negative in most cases. 

**Figure 4:**
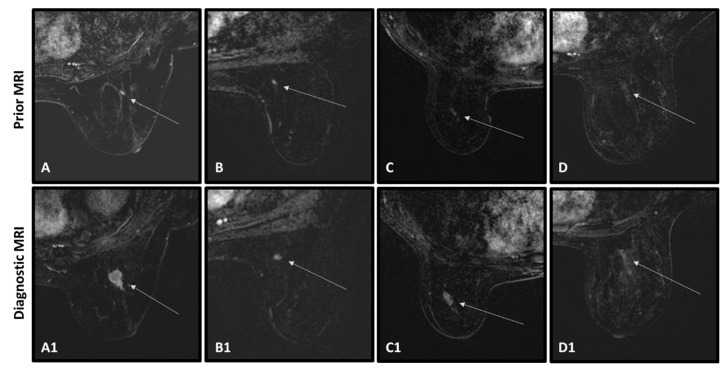
Four cancer cases of a successful classification of the AI network based on the prior MRIs. In all cases, an abnormality was retrospectively found in the prior scans but was not suspected at the time. Top row shows the prior MRIs of the patients (**A**–**D**), arrow pointing to an enhancing abnormality. Bottom row shows the ‘diagnostic’ MRIs of the patients (**A1**–**D1**), arrows pointing to the diagnosed cancerous tumors.

**Figure 5:**
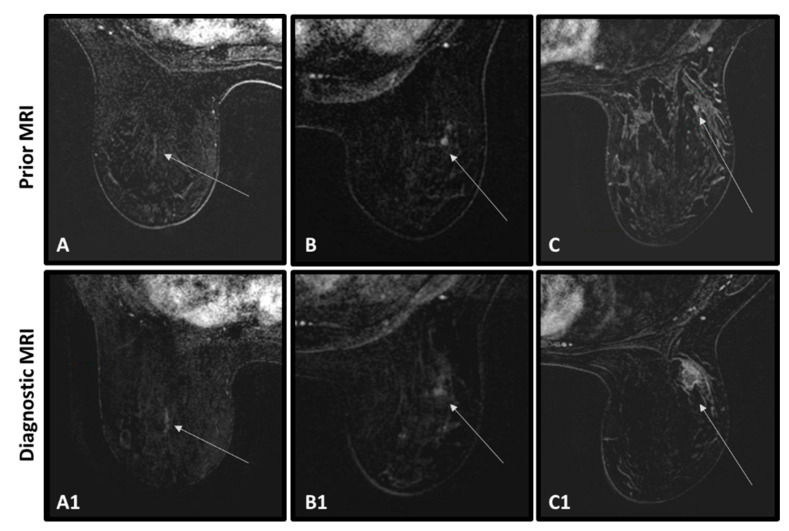
Three cancer cases of an unsuccessful classification of the AI network. In all cases, an abnormality was retrospectively found in the prior scans but was not suspected at the time by the radiologist. Top row shows the prior MRIs of the patients (**A**–**C**), arrow pointing to an enhancing abnormality. Bottom row shows the ‘diagnostic’ MRIs of the patients (**A1**–**C1**), arrows pointing to the diagnosed cancerous tumors.

**Figure 6:**
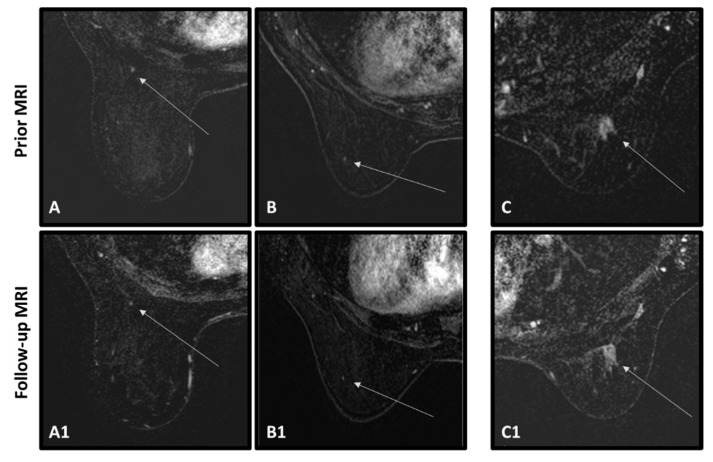
Two benign cases of a successful classification of the AI network (**A**,**A1**,**B**,**B1**) and one benign case of an unsuccessful classification of the AI network (**C**,**C1**). In all cases, an abnormality was retrospectively found in the prior scans. Top row shows the prior MRIs of the patients, arrow pointing to an enhancing abnormality. Bottom row shows the follow-up MRIs of the patients, arrows pointing to the benign tumors.

Representative cases of successful and unsuccessful classification of the AI algorithm in cancerous and benign lesions are shown in Figure 4, Figure 5 and Figure 6 (distinct from the cases presented in Figure 1). In Figure 4, four successful classifications of cancer tumors are seen, showing the abnormalities that appeared in the prior MRIs (top row) and the detected tumors in the diagnostic MRIs (bottom row). In Figure 4A,B, the abnormalities were detected at the time of the prior MRIs by the radiologist, but were said to be post-surgical changes and given BI-RADS 3. Both were diagnosed ~1 year later by the ‘diagnostic’ MRI as IDCs. In Figure 4C,D, there was no suspicious radiological finding at the time of the prior MRIs. The abnormalities were noted only retrospectively. Both were diagnosed by the ‘diagnostic’ MRIs as in-situ carcinomas. Figure 5 shows three examples of unsuccessful classifications of cancerous tumors. All were given BI-RADS 2 in the prior MRIs (Figure 5A–D) and the radiological abnormalities were only retrospectively detected. Note that, in Figure 5A,C, the abnormalities are mildly conspicuous and particularly difficult to differentiate from the enhancing background. 

Figure 6 shows three benign cases. Two of them were correctly classified by the AI algorithm and one was not. The correctly classified lesions were radiologically detected in the prior MRIs, one was given BI-RADS 0 and the patient’s previous MRI was requested to observe whether the lesion had already been present (Figure 6A), and the other was given BI-RADS 2 because the lesion was known and seemed unchanged (Figure 6B). The following MRIs of both cases were given BI-RADS 2. The incorrectly classified case (Figure 6C) was given BI-RADS 4 due to non-mass enhancement that appeared in the prior scan. The patient was recommended to complete more imaging tests and a biopsy, but the lesion was not detected under targeted ultrasound (US) nor a following MRI, therefore was finally determined as parenchymal enhancement. A follow-up MRI, almost 1 year later, showed sporadic enhancement areas which were defined as BPE, and the MRI was given BI-RADS 2. 

## 4. Discussion

In this study, we challenged an AI-based network by investigating its ability to classify sub-centimeter breast abnormalities that were not originally suspected by the radiologist. As shown herein, breast abnormalities that are localized to the same anatomical area where the tumor was diagnosed on subsequent MRI can be visualized on initial MRIin ~60% of the cases. These results are in line with previously published studies [17,18,19,20]. Korhonen et al. [15] summarized the main reasons for false-negative errors: (1) technical—patient motion, artifacts etc. (2) perceptual—poor lesion conspicuity, subtle appearance of lesion etc. (3) cognitive—wrong interpretation. Misinterpretation of lesions was also found to increase the false-negative errors, mainly due to the presence of multiple breast lesions, prior biopsy or surgery and stability in size [17,18]. Bilocq-Lacoste et al. [17] reported that cancers that were undiagnosed had no specific MRI characteristics, receptor status, or risk factors, such as gene mutation, chest radiation, family history and site of previous biopsy. Most of the undiagnosed cancers (51 from 77, 66%) were overlooked due to their small size and high BPE. In agreement with Bilocq-Lacoste et al. [17], in the current study, the abnormalities in the prior scans were significantly smaller than the diagnosed tumors.

Clauser et al. [38] reported that the rate of small foci (<0.5 cm lesions) identified by MRI in a high-risk population was 31.3% (from a total of 166 patients). An automated approach based on CAD was reported in 2016 [20], where 71% of prior visible lesions and 31% of prior minimally visible lesions were detected in a group of 40 high risk cases. Several studies evaluated additional tools for an improved characterization of sub-centimeter breast lesions detected by breast imaging. Gibbs et al. [39] showed that radiomic analysis of small breast lesions is feasible; they showed significant differences between benign and malignant lesions for 53/133 calculated features, with high negative (>89%) and positive (>83%) predictive values. Lo Gullo et al. [40] showed that the combination of radiomics and machine learning improves the differentiation between benign and malignant small breast lesions in *BRCA* PV carriers compared with the BI-RADS classification by the radiologists. The integration of AI algorithms with the Internet-of-Things (IoT) has the potential to drive substantial advancement in the diagnosis of early stage breast cancer. In recent years, IoT has emerged as a revolutionary technology in healthcare. For example, it has been reported as a breakthrough in the surgical field [41]. Its ability to securely transmit, store and analyze data in real-time may assist radiologists in detection and characterization of breast lesions with increased efficiency and accuracy. Peta et al. [42] proposed an IoT-based model using data from bio-thermal sensors for the classification of breast cancer. The model achieved an accuracy of above 90%. Majji et al. [43] used mammograms and IoT for classification of breast lesions. Here too, the model achieved a high accuracy of above 90%. In this study, an AI-based network could accurately re-classify more than half of cancerous breast MRI abnormalities in *BRCA* PV carriers with a low rate of false-positives. Notably, triple-negative tumors were more frequently successfully classified as cancerous compared with other histological tumor types. This may be related, in all likelihood, to the fact that *BRCA1* mutated cases represented 85.7% of the successfully classified group and to the well-established association of triple-negative breast tumors with specific MRI features [44,45,46]. In a study by Moffa et al. [45], the majority of the triple-negative tumors appeared as regular shaped mass enhancements (round or oval) with circumscribed margins by MRI. A large portion of triple-negative breast cancers also presented with a rim enhancement which was shown to be a positive predictor of this subtype. Importantly, triple-negative is associated with an increased angiogenesis and with a higher recurrence rate [45]. Therefore, the ability to detect triple-negative breast cancer at such an early stage, while abnormalities are mostly foci with no rim-enhancement, may reduce the clinical burden of the disease. 

There were several limitations in this study that need to be acknowledged: a small cohort size, the use of one 2D central tumor slice instead of the whole 3D volume and no age-matched cancer-free control patients. Yet the limited spectrum of germline *BRCA* PV, the minimal inter-observer variability (due to the fact that all were carried out in a single medical center using the same staff and equipment), and the availability of longitudinal data on study participants are notable advantages. 

## 5. Conclusions

In this study, we presented the ability to accurately classify sub-centimeter MRI-detected breast abnormalities that were not suspected by the radiologist and were subsequently diagnosed as cancer at an average of 1 year later. Future work includes applying such a classification on larger groups of patients in a prospective manner and broadening the AI-network to an automatic detection of suspicious abnormalities. If successful in these additional studies, such an approach can potentially facilitate earlier BC detection in high-risk women.

## Figures and Tables

**Table 1:** Radiological features and pathological characteristics of study participants.

*Characteristics*	*All Cancer Patients*	*Cancer Patients with Abnormality*	*Cancer Patients no Abnormality*	*p Value Cancer with/without Abnormality*	*All Cancer-Free Patients*	*p Value Cancer/Cancer-Free*
*Number of patients*	53	32 (60.4)	21 (39.6)		53	
*Age at diagnosis (range-years)*	52 ± 14 (32–80)	51.4 ± 13.6 (33–78)	51.9 ± 14.4 (32–80)	0.96	50 ± 15.9 (23–78)	0.71
*Mutated gene*				0.70		**0** **.015**
*BRCA1*	39 (73.6)	25 (78.1)	14 (66.7)		25 (47.2)	
*BRCA2*	14 (26.4)	7 (21.9)	7 (33.3)		26 (49.1)	
*unknown*					2 (3.8)	
*Days between scans (range-days)*	367.6 ± 130.2 (177–938)	361 ± 118.6 (177–779)	380 ± 148.2 (182–938)	0.68	373 ± 78.5 (177–604)	0.78
*BIRADS on prior scan*				0.44		0.28
*0*	7 (13.2)	5 (15.6)	2 (9.5)		4 (7.6)	
*1*	2 (3.8)	2 (6.3)	--		5 (9.4)	
*2*	33 (62.3)	17 (53.1)	16 (76.2)		33 (62.3)	
*3*	11 (2.8)	8 (25)	3 (14.3)		8 (15.1)	
*4*	--	--	--		3 (5.7)	
*BPE on prior scan*				0.08		0.09
*minimal-mild*	31 (58.5)	16 (50)	15 (71.4)		40 (75.5)	
*moderate-marked*	22 (41.5)	16 (50)	6 (28.6)		13 (24.5)	
*Tumor size at diagnosis [mm]*	10.8 ± 7.3 (2–35)	12.8 ± 8.4 (2–35)	7.8 ± 4 (3–16)	**0.01**		
*Tumor type*				0.77		
*IDC*	33 (62.3)	18 (56.3)	15 (71.4)			
*DCIS*	17 (32.1)	12 (37.5)	5 (23.8)			
*IDC+DCIS*	2 (3.8)	1 (3.1)	1 (4.8)			
*unknown*	1 (1.9)	1 (3.1)	--			
*Histological grade*				0.36		
*Low*	2 (3.8)	1 (3.1)	2 (9.5)			
*Intermediate*	14 (26.4)	8 (25)	6 (28.6)			
*High*	33 (62.3)	19 (59.4)	14 (66.7)			
*unknown*	4 (7.6)	4 (12.5)	--			
*Luminal type*				0.35		
*HR+/HER2−*	18 (33.9)	10 (31.3)	8 (38.1)			
*HR+/HER2+*	3 (5.7)	2 (6.3)	1 (4.8)			
*HR−/HER2+*	4 (7.6)	1 (3.1)	1 (4.8)			
*Triple negative*	30 (56.6)	19 (59.4)	11 (52.4)			

Continuous data: mean ± SD, with ranges in parenthesis. Categorical data: number of patients with percentages in parenthesis. IDC = invasive ductal carcinoma, DCIS = ductal carcinoma in situ, HER2 = human epidermal growth factor receptor, HR = hormone receptor.

**Table 2:** Lesion characteristics at diagnosis and in the prior scans.

		*Cancerous Lesions*		*Non-Cancerous Lesions/BPE*		*Time*		*Interaction Time × Group*		*Paired t-Test*	
		*Prior Scan (N = 32)*	*At Diagnosis (N = 32)*	*Prior Scan (N = 53)*	*Follow-Up Scan (N = 33)*	*F*	*p*	*F*	*p*	*Cancerous Lesions p*	*Non-Cancerous Lesions p*
*Lesion size [mm]*		6.1 ± 4.2 (1.5–17)	10.8 ± 7.3 (2–35)	7.4 ± 4.2 (1.7–27.3)	7.1 ± 5.1 (2.3–28.9)	F(1,59) = 23.74	**<0.0001**	F(1,59) = 25.99	**<0.001**	**<0.0001**	0.69
*Morphology*						F(1,63) = 5.96	**0.02**	F(1,63) = 13.87	**<0.001**	**0.0001**	0.37
	*Focus*	19 (59.3)	6 (18.8)	12 (22.6)	12 (36.4)						
	*Mass*	6 (18.8)	18 (56.3)	17 (32.1)	10 (30.3)						
	*Non-mass*	7 (21.8)	8 (25)	25 (47.2)	11 (33.3)						
*Kinetics*											
	*CAD*	9 (28.1)	14 (43.8)	4 (7.6)	1 (3)	F(1,61) = 0.93	0.34	F(1,61) = 4.78	**0.03**	0.09	0.16
*Initial phase*						F(1,61) = 0.41	0.53	F(1,61) = 1.55	0.22		
	*Slow*	8 (25)	5 (15.6)	24 (45.3)	16 (48.5)						
	*Medium*	13 (40.6)	13 (40.6)	15 (28.3)	8 (24.2)						
	*Fast*	11 (34.4)	14 (43.8)	14 (26.4)	9 (27.3)						
*Delayed phase*						F(1,62) = 2.11	0.15	F(1,62) = 0.24	0.75		
	*Persistent*	23 (71.9)	21 (65.6)	45 (84.9)	24 (72.7)						
	*Plateau*	8 (25)	10 (31.3)	7 (13.2)	7 (21.2)						
	*Washout*	1 (3.1)	1 (3.1)	1 (1.9)	2 (6.1)						

Continuous data: means, with ranges in parenthesis. Categorical data: numbers of patients with percentages in parenthesis.

**Table 3:** Radiological and pathological characteristics of successfully and unsuccessfully classified abnormalities in prior scans of cancer patients.

Characteristics		AI Success (21)	AI Failure (11)	*p*-Value
*Age at diagnosis (range-years)*		52.5 ± 13.4 (34–77)	49.3 ± 14.3 (33–78)	0.53
*Mutated gene*				0.39
	*BRCA1*	18 (85.7)	7 (63.6)	
	*BRCA2*	3 (14.3)	4 (36.4)	
*Days between scans (range-days)*		362.7 ± 89.3 (196–511)	357.7 ± 166.1 (177–779)	0.85
*BIRADS on prior scan*				1
	0	4 (19.1)	1 (10)	
	1	1 (4.8)	1 (10)	
	2	11 (52.4)	6 (60)	
	3	5 (23.8)	2 (20)	
*BPE on previous scan*				0.57
	Minimal to mild	12 (57.1)	5 (45.5)	
	Moderate to marked	9 (42.9)	6 (54.5)	
*Tumor size [mm]*		10.6 ± 8 (2–28)	16.7 ± 7.8 (7–35)	**0.05**
*Tumor type*				0.42
	IDC	13 (61.9)	5 (45.5)	
	DCIS	6 (28.6)	6 (54.5)	
	IDC+DCIS	1 (4.8)	--	
	unknown	1 (4.8)	--	
*Histological grade*				0.68
	Low	0	--	
	Intermediate	4 (19.1)	4(40)	
	High	14 (66.7)	5(50)	
	unknown	3 (14.3)	1(10)	
*Molecular subtype*				**0.016**
	HR+/HER2-	5 (23.8)	5 (50)	
	HR+/HER2+	1 (4.8)	--	
	HR-/HER2+	2 (9.5)	2 (20)	
	Triple negative	13 (61.9)	3 (30)	

**Table 4:** Lesion characteristics of successfully and unsuccessfully classified abnormalities in prior scans of cancer patients.

Characteristics of Early Scan		AI Success (21)	AI Failure (11)	*p*-Value
*Morphology*				1
	focus	12 (57.1)	7 (63.6)	
	mass	4 (19.1)	2 (18.2)	
	non-mass	5 (23.8)	2 (18.2)	
*Initial enhancement*				0.71
	Slow	5 (23.8)	2 (18.2)	
	Medium	8 (38.1)	6 (54.5)	
	fast	8 (38.1)	3 (27.3)	
*Delayed phase*				0.47
	Persistent	15 (71.4)	8 (72.7)	
	Plateau	6 (28.6)	2 (18.2)	
	washout	--	1 (9)	
*CAD*				1
	Positive	6 (28.6)	3 (27.3)	
	negative	13 (61.9)	8 (72.7)	
	unknown	1 (4.8)		

Abbreviation: CAD, computer aided detection.

## Data Availability

The data that support the findings of this study are available from the corresponding author, D.A., upon reasonable request.
